# Artificial Intelligence and Technology in Aging

**DOI:** 10.1093/geroni/igaf122.1613

**Published:** 2025-12-31

## Abstract

This session provides insight into the use of artificial intelligence in disease and functional assessments.

